# Northern refugia and recent expansion in the North Sea: the case of the wrasse *Symphodus melops* (Linnaeus, 1758)

**DOI:** 10.1002/ece3.77

**Published:** 2012-01

**Authors:** Joana I Robalo, Rita Castilho, Sara M Francisco, Frederico Almada, Halvor Knutsen, Per E Jorde, Ana M Pereira, Vitor C Almada

**Affiliations:** 1 Unidade de Investigação em Eco-Etologia, ISPA—Instituto UniversitárioRua Jardim do Tabaco 34, 1149-041 Lisboa, Portugal; 2 Centro de Ciências do Mar (CCMAR, CIMAR-Associate Laboratory), Universidade do AlgarveCampus de Gambelas, 8005-139 Faro, Portugal; 3 Departamento de Zoologia e Antropologia, Faculdade de Ciências da Universidade do PortoPraça Gomes Teixeira, 4099-002 Porto, Portugal; 4 Institute of Marine Research (IMR), Flødevigen Marine Research StationHis, Norway; 5 Centre for Ecological and Evolutionary Synthesis (CEES), Department of Biology, University of OsloBlindern, Oslo, Norway; 6 University of Agder4604 Kristiansand, Norway; 7 CIMAR/CIIMAR, Oporto UniversityRua dos Bragas 289, 4050-123 Porto, Portugal

**Keywords:** Atlantic coast, glacial refugia, Labridae, North Sea, phylogeography, spatial variation of genetic diversity

## Abstract

Pleistocene climate changes have imposed extreme conditions to intertidal rocky marine communities, forcing many species to significant range shifts in their geographical distributions. Phylogeographic analyses based on both mitochondrial and nuclear genetic markers provide a useful approach to unravel phylogeographic patterns and processes of species after this time period, to gain general knowledge of how climatic changes affect shifts in species distributions. We analyzed these patterns on the corkwing wrasse (*Symphodus melops*, Labridae), a rocky shore species inhabiting North Sea waters and temperate northeastern Atlantic Ocean from Norway to Morocco including the Azores, using a fragment of the mitochondrial control region and the first intron of the nuclear S7 ribosomal protein gene. We found that *S. melops* shows a clear differentiation between the Atlantic and the Scandinavian populations and a sharp contrast in the genetic diversity, high in the south and low in the north. Within each of these main geographic areas there is little or no genetic differentiation. The species may have persisted throughout the last glacial maximum in the southern areas as paleotemperatures were not lower than they are today in North Scandinavia. The North Sea recolonization most likely took place during the current interglacial and is dominated by a haplotype absent from the south of the study area, but present in Plymouth and Belfast. The possibility of a glacial refugium in or near the English Channel is discussed.

## Introduction

A major concern in studies of phylogeography of northeastern Atlantic organisms has been the location of warm water refugia where temperate fish survived the successive glacial peaks of the Pleistocene (the most recent of which ended about 10–11,000 years before present) and from where the recolonization of more northerly areas took place when temperatures arose in the interglacials (e.g., Wares and Cunningham 2001). Regarding the last glaciation, for which paleoclimatic data are available in greater detail, it is known that the North Sea was basically glaciated loosing most habitats suited for marine organisms (e.g., CLIMAP 1981; Hayes et al. 2005). The polar front advanced to the south reaching the western shores of the Iberian Peninsula (Dias et al. 1997) and even the Mediterranean was several degrees cooler than today (e.g., Thiede 1978). In this context, the warm temperate fish fauna typical of the Lusitanian province (sensu Briggs 1995, which extends from the English Channel to the Cape Verde in West Africa, including the Mediterranean and the Black Sea, and also Azores, Madeira, and Canaries), could have only survived in areas that retained warmer water pockets (Briggs 1995). These warmer areas are known to have existed inside the Mediterranean (Thiede 1978), in some of the North Atlantic archipelagos and in the eastern Atlantic, probably from South Iberia to the south (CLIMAP 1981). At the peak of the last glaciation (Last Glacial Maximum, LGM, about 18,000 years ago), suitable conditions for the more cold tolerant species, such as *Symphodus melops* (Linnaeus, 1758), seem to have existed in broad areas of the Mediterranean and in the Atlantic, from central Portugal southwards (CLIMAP 1981; NOAA 2006). Indeed, if we compare the temperatures provided for the LGM by CLIMAP (February and August) with those provided by NOAA for the data series 1982–2007, it is clear that, from central Portugal to the south, summer temperatures in the LGM were comparable to those now prevailing in Scandinavia, an area were the species is known to breed successfully. In the winter, the sea surface temperatures from central Portugal southwards were, in the LGM, around 6–12°C. This is a milder condition compared to present winter temperatures in Scandinavia (3–8°C) (CLIMAP 1981; NOAA 2006).

Recent studies on the phylogeography of West European marine organisms often attempted to address the following issues: (1) Did warm temperate organisms of western Europe survived in refugia inside the Mediterranean and recolonized the north east Atlantic after the end of the last glaciation, about 10,000 years ago (e.g., Almada et al. 2001; Domingues et al. 2005)? (2) Did marine populations follow the classical phylogeographic patterns of terrestrial species (e.g., Hewitt 1999, 2000, 2004; Avise 2000) with a marked decline of diversity to the north assumed to result from a succession of bottlenecks, caused by the fact that each northward advance likely included a limited sample of the genetic variants present to the south (e.g., Avise 2000). The populations at the northern limits of a species range should display very low numbers of genetic variants, often with a few alleles that in other places are not abundant reaching dominance because of the stochastic effects of drift (“gene surfing,”Excoffier and Ray 2008; Hallatschek and Nelson 2008; Excoffier et al. 2009).

In this respect, the phylogeographic studies of West European marine organisms have yielded mixed results. Some studies demonstrate the role of the Mediterranean as a refugium and source of recolonization of the Atlantic (e.g., Domingues et al. 2005, 2008). However, several other studies highlighted a number of unresolved issues. For some species, there are now soft-barriers to gene flow between the Mediterranean and adjacent Atlantic (for a review, see Patarnello et al. 2007). If these soft-barriers operated in the beginning of the current interglacial, they would act against the expansion of the Mediterranean fish to the north. Some studies confirmed a very marked drop of genetic diversity in the north of a species range, but the species or lineage is not present in the Mediterranean (often being replaced by a Mediterranean sister clade), raising also doubts on the role of the Mediterranean as a refugium (e.g., Domingues et al. 2007; Francisco et al. 2009). A third group of cases includes organisms that show high connectivity throughout their entire ranges from the Mediterranean to the north, with no clear drop of diversity in the extreme north (e.g., *Homarus gammarus*Triantafyllidis et al. 2005; *Nephrops norvegicus*, Stamatis et al. 2004; *Sprattus sprattus*, Debes et al. 2008; *Lipophrys pholis*, Francisco et al. 2011). Finally, for some organisms, the evidence points to refugia inside the North Sea or at its vicinity (e.g. Provan et al. 2005; Triantafyllidis et al. 2005). Thus, the overall phylogeographic pattern of the northeastern Atlantic hardly fits a single explanation.

This diversity of phylogeographic patterns means that more species must be studied if we are to understand the relative importance of each pattern and identify ecological and life-history traits and historical processes that can generate them.

In the present paper, we use one mitochondrial and one nuclear marker to assess the phylogeographic patterns and processes of *S. melops* with samples ranging from South Portugal to Norway and Sweden. We aim to find out if there was evidence of post glacial expansion and identify potential or probable refugia from where recolonization took place. Such questions will shed light upon how coastal species with low dispersal potential were affected by temperature changes in this time period.

## Material and Methods

### Study species

*Symphodus melops* is an interesting species that can add to our understanding of the patterns of recolonization of West European shores after the last glaciation. It is a rocky shore species inhabiting temperate Atlantic waters from Norway to Morocco and the Azores (e.g., Froese and Pauly 2011). The adults have little dispersal ability and probably spend their postsettlement life in a limited stretch of rocky shore (Lejeune 1985), thus dispersal must be achieved almost exclusively by the planktonic larvae. Eggs are demersal and are guarded by the males in the nest until hatching of the larvae (Potts 1984). At least two studies addressed the phylogeny of the genus *Symphodus* (Almada et al. 2002; Hanel et al. 2002). Both studies converged in the redefinition of the composition of *Symphodus*, which after these changes corresponds to a solid monophyletic clade. The genus *Symphodus* displays the highest number of species in the Mediterranean (Froese and Pauly 2011) with species diversity rapidly decreasing to the north and south of Gibraltar strait. Only *S. melops* and *S. bailloni* reach the North Sea, and there are no tropical members of the genus. Thus, the clade is a typical warm temperate group. *Symphodus melops* is rare within the Mediterranean (where it is replaced by another *Symphodus* species, *S. roissali*, Almada et al. 2002; Hanel et al. 2002), so its distribution extends in latitude mainly from Portugal to Scandinavia. In the British Isles and Scandinavia, this species is often fished for use as a “cleaner” in salmon culture facilities (e.g., Knutsen and Sannæs 2009), so the genetic assessment of the populations and their relationships with those more to the south is of great importance for the proper conservation and management of this fish.

### Sampling

**Figure 1:**
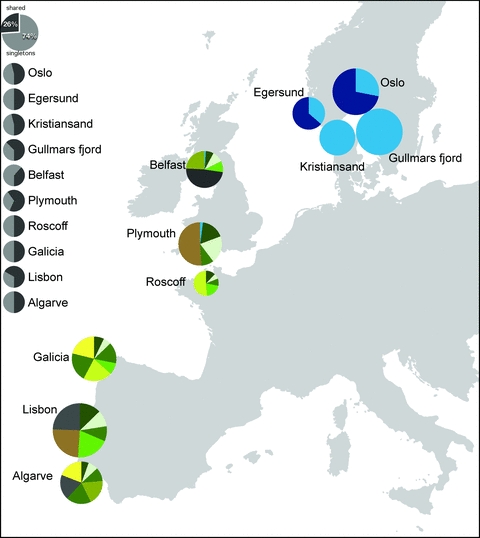
Map with the sampling locations. Top gray circle represents proportion of singleton haplotypes (light gray) and shared between individual haplotypes (dark gray), while smaller gray circles represent the same proportion for each location. Colored circles represent only the relative proportion of shared between individual haplotypes in each location, with each haplotype having a different color. Size of colored circle is proportional to sample size.

Samples of *S. melops* were collected by beach seining and/or spear fishing from 10 locations along West Europe, from south of Portugal to Norway (Fig. 1). These included Algarve and Lisbon (Portugal), Galicia (Spain), Roscoff (France), Plymouth (United Kingdom), Belfast (Ireland), Gullmars fjord (Sweden), Oslo, Kristiansand, and Egersund (Norway). A total of 263 individuals were obtained, and the number of samples collected per site is listed in Table 1.

**Table 1:** Sampling locations, haplotypes per site, and diversity measures for *Symphodus melops* mitochondrial control region (CR) sequences. *N* = number of individuals per location, NH = number of haplotypes

Location	Country	*N*	NH	Haplotype diversity ± SD	Nucleotide diversity ± SD	Mean number of pairwise differences
Algarve	Portugal	27	13	0.83 ± 0.07	0.55 ± 0.36%	2.0 ± 1.2
Galicia	Spain	29	13	0.79 ± 0.07	0.48 ± 0.32%	1.8 ± 1.1
Plymouth	UK	28	13	0.78 ± 0.08	0.70 ± 0.43%	2.5 ± 1.4
Belfast	Ireland	24	9	0.78 ± 0.08	0.63 ± 0.40%	2.3 ± 1.3
Roscoff	France	16	8	0.70 ± 0.13	0.34 ± 0.26%	1.3 ± 0.8
Lisbon	Portugal	35	12	0.67 ± 0.09	0.43 ± 0.29%	1.6 ± 1.0
Oslo	Norway	30	4	0.25 ± 0.10	0.07 ± 0.09%	0.3 ± 0.3
Egersund	Norway	21	3	0.19 ± 0.11	0.05 ± 0.08%	0.2 ± 0.3
Kristiansand	Norway	23	2	0.09 ± 0.08	0.02 ± 0.05%	0.1 ± 0.2
Gullmars fjord	Sweden	30	2	0.07 ± 0.06	0.02 ± 0.04%	0.1 ± 0.1

### DNA extraction, amplification, and sequencing

Total genomic DNA was extracted from fin or muscle samples preserved in 96% ethanol with the REDExtract-N-Amp kit (Sigma-Aldrich Corporation, http://www.sigmaaldrich.com/) following the manufacturers instructions. Voucher specimens are deposited in ISPA (ethanol preserved tissues). PCR amplification of mitochondrial control region (CR) and the first intron of the nuclear S7 ribosomal protein gene (S7) was performed with the following pairs of primers: dloop—LPro1 (5′-ACTCT CACCC CTAGC TCCCA AAG-3′) and HDL1 (5′-CCTGA AGTAG GAACC AGATG CCAG-3′) (Ostellari et al. 1996) and the first intron of the S7 ribosomal protein gene—S7RPEX1F (5′-TGG CCT CTT CCT TGG CCG TC-3′) and S7RPEX2R (5′-AAC TCG TCT GGC TTT TCG CC-3′) (Chow and Hazama, 1998). PCR amplification reactions were performed in a 20-µl total-reaction volume with 10 µl of REDExtract-N-ampl PCR reaction mix (Sigma – Aldrich Corporation, http://www.sigmaaldrich.com/), 0.8 µl of each primer (10 µM), 4.4 µl of Sigma-water, and 4 µl of template DNA. An initial denaturation at 94°C for 7 min was followed by 35/30 cycles (denaturation at 94°C for 30/45 sec, annealing at 55°C for 30/45 sec, and extension at 72°C for 1 min; values CR/S7, respectively) and a final extension at 72°C for 7 min on a BioRad Mycycler thermal cycler. The same primers were used for the sequencing reaction, and the PCR products were purified and sequenced in STABVIDA (http://www.stabvida.net/).

### DNA analysis

All sequences were aligned using Clustal X (Thompson et al. 1997).

ARLEQUIN software package v.3.5 (Excoffier and Lischer 2010) was used to estimate the genetic diversity within each sample, to access potential population differentiation, and to perform neutrality tests. It was also used to perform analysis of molecular variance (AMOVA; Excoffier et al. 1992) and to compute pairwise *F*_ST_s. Significance levels of all multiple statistical tests were corrected using the false discovery rate (FDR) approach (Benjamini and Hochberg 1995) implemented in QVALUE package of software R (Dabney et al. 2006). In the case of the S7 intron, the analyses were also run in ARLEQUIN, after allowing the program to reconstruct the haplotypes present, using the ELB algorithm (Excoffier et al. 2003).

For the CR, the spatial analysis of molecular variance (SAMOVA 1.0) (Dupanloup et al. 2002) was used to identify groups of sampling locations that are geographically and genetically homogeneous and maximally differentiated from each other. This approach relies on a technique of AMOVA (Excoffier et al. 1992). However, in contrast to conventional AMOVA, SAMOVA does not require that the groups’ constitution is defined a priori, allowing instead the groups to emerge from the data. The most likely number of groups was identified by running SAMOVA with two to nine groups and choosing the partition scheme with the highest *F*_CT_ value.

Because SAMOVA identified two groups that maximized *F*_CT_ (see the results section), the data of the populations included in each group were pooled and mismatch analysis (Rogers and Harpending 1992; Rogers 1995), and Fu's *F*s (Fu 1997) and Tajima's *D* (Tajima 1983) tests were performed to test for possible bottlenecks and population expansion in each group. The χ^2^-test developed by Salicru et al. (1993) was used to access the significance of the differences of haplotype diversity values among populations. To estimate nucleotide pairwise genetic distances between locations, we used Nei and Li (1979) distance, as implemented in ARLEQUIN, to correct for inherited ancestral polymorphism.

A haplotype network was constructed for the CR data using Network v.4.5 (Bandelt et al. 1999). Due to the high gene diversity in the mtDNA CR, the dataset was first reduced using the star contraction preprocessing (Forster et al. 2001) that identifies and contracts star-like phylogenetic clusters of sequences into single representative sequences. Median networks that contained all possible equally short trees were simplified by running the maximum parsimony (MP) calculation option to eliminate superfluous nodes and links (Polzin and Daneschmand 2003).

Past population demography of *S. melops* was inferred with the CR data using both mismatch distributions and the linear Bayesian skyline plot (BSP) (Drummond et al. 2005) model as implemented in BEAST v.1.6 (Drummond and Rambaut, 2007) employing the Bayesian MCMC (Markov chain Monte Carlo)coalescent method, an HKY + I + G model of substitution, and a strict clock. The Bayesian distribution was generated using results from five independent runs of 150 million MCMC steps obtaining effective samples sizes (ESS) of parameter estimates of over 200, with a burn-in of 10%. The time for most recent common ancestor (*t*_MRCA_) and the median and corresponding credibility intervals of the BSP were depicted using Tracer v.1.4 (Rambaut and Drummond 2007). mtDNA CR mutation rates in fish are widely variable, 2.2–4.5%/ Million Years (MY) between lineages for East African cichlids (Cichlidae, Sato et al. 2003), 3.6%/MY for snooks (Centropomidae, Donaldson and Wilson 1999), 15–20%/MY for Indo-Pacific sardines (Clupeidae, Bowen and Grant 1997). In the absence of a clock calibration for the CR of *S. melops*, we address the uncertainty by tentatively assuming a within-lineage mutation rate of 5%/MY. This figure was based on previously published work on several fish species that point to the between-lineage divergences of the CR to be about five times higher than the comparable divergences with mtDNA cytochrome *b*. This gene has been calibrated in multiple bony fish at about 1.2–2%/MY (Doadrio and Perdices 2005; Bowen et al. 2001; Bermingham et al. 1997). For the mismatch analysis, we also present results assuming within-lineage per site mutation rate of 3.5%/MY and 10%/MY to place the 5% value in a broader context. These values are within the range of values found by Bowen et al. (2006) after a review of CR molecular clock calibrations for several tropical Atlantic fish species. These estimations of divergence times must be interpreted with great caution. Apart from uncertainties in the molecular clock calibrations, they often assume a model of mutation–drift equilibrium that does not hold if severe bottlenecks occurred. If two sister lineages differ markedly in their population size, the one with smaller size will change at a faster rate due to increased drift. In this situation, divergence times between the two populations will be overestimated.

Migration rates among populations were estimated by the MCMC approach implemented in Lamarc v. 2.1.3 (Kuhner 2006), using 10 runs of 12 short chains of 1000 steps and five long chains of 50,000 steps, with a burn-in of 10,000.

## Results

The CR dataset consisted of a total of 364 characters (263 individuals, Genbank Accession Numbers from JN035910 to JN036172), representing a total of 50 haplotypes. The amplification of the first intron of the S7 gene yielded a fragment of 520 base pairs, with 10 SNPs (197 individuals, Genbank Acession Numbers from JN036173 to JN036369). Only two samples from Belfast were amplified for the S7 gene, thus this population was excluded from the subsequent analyses.

**Figure 2:**
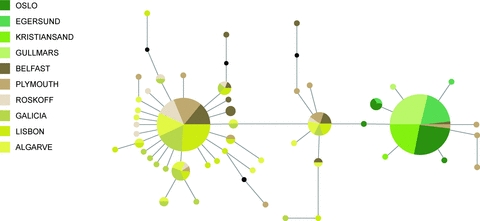
Median joining tree of the haplotypes detected in *Symphodus melops*. The area of the circles is proportional to each haplotype frequency. Colors refer to the region in which haplotypes were found. In the case where haplotypes are shared among regions, shading is proportional to the frequency of the haplotype in each region.

The CR haplotype network shows two dominant haplotypes, separated by two mutational steps, with low frequency of rare alleles connected by only a few mutational steps (Fig. 2). These two clades display a clear geographic structure. The more ramified group includes fish from central and south Portugal, Galicia, Roscoff, Plymouth, and Belfast. No fish from Scandinavia belong to this group. The second group contains all fish from Scandinavia and some fish from Plymouth and Belfast. The difference between the two groups is very clear-cut. While the first group contains many derived haplotypes often separated by several mutational steps from the main haplotype, in the smaller group the level of ramification is much more restricted. Thus, the two groups represent conditions of high and low genetic diversity, respectively.

**Figure 3:**
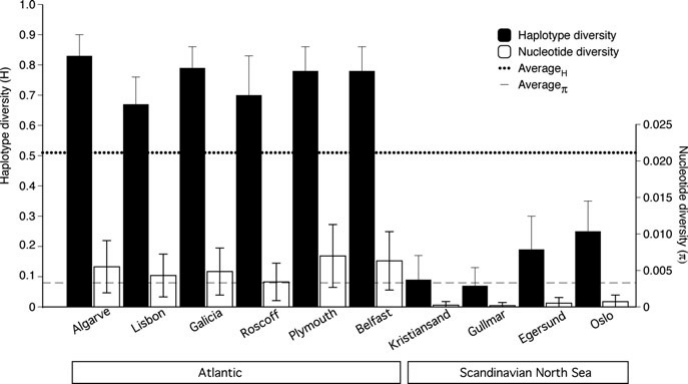
Haplotype diversity and nucleotide diversity across all populations sampled.

For the CR fragment, the genetic diversity indices for each population are summarized in Table 1 (see also Fig. 3). The diversity indices for S7 are presented in Table 2. The Atlantic populations are much more diverse than the ones from Scandinavia for all indices and for both markers with the exception of the sample from Oslo in the S7 gene. The pairwise *F*_ST_s and corrected average pairwise differences are presented in Tables 3 (CR) and 4 (S7). For both markers, all Scandinavian populations display *F*_ST_s and mean numbers of corrected pairwise differences that are not significant among themselves. They are all significantly different from the non-Scandinavian populations, except for the *F*_ST_ between Oslo and Roscoff for the S7 gene. Another cluster of populations that do not differ significantly are those from South and Central Portugal and Galicia. The populations of Belfast, Plymouth, and Roscoff occupy a somewhat intermediate position in the CR. Indeed, for the CR, while the Iberian cluster (Portugal and Galicia) displays significant *F*_ST_s and corrected average pairwise differences with Plymouth and Scandinavian populations, the populations of Roscoff and Belfast do not display significant differences when compared with Plymouth, also failing to display significant differences when compared with the Portuguese and Galician populations. For the S7, all populations from South Portugal to Plymouth display no significant differences (Belfast could not be included due to low sample size for this marker).

**Table 2:** Sampling locations, haplotypes per site, and diversity measures for *Symphodus melops* S7 first intron. *N* = number of individuals per location, NH = number of haplotypes (as calculated by Arlequin with the EBL algorithm), Average number of pairwise differences within populations and FIS (inbreeding coefficient). Significant values in bold (*P* < 0.05)

Location	Country	N	NH	Nucleotide diversity ± SD	Average number of pairwise differences within populations	FIS FIS
Algarve	Portugal	19	12	0.33 ± 0.22%	1.550	0.189
Lisbon	Portugal	25	28	0.30 ± 0.20%	1.639	0.000
Galicia	Spain	28	14	0.32 ± 0.21%	1.458	−0.104
Roscoff	France	13	12	0.34 ± 0.26%	1.397	−0.222
Plymouth	UK	26	17	0.39 ± 0.24%	1.758	0.149
Oslo	Norway	26	12	2.99 ± 1.50%	15.103	0.010
Egersund	Norway	19	6	0.19 ± 0.15%	0.509	**0.489**
Kristiansand	Norway	15	8	0.17 ± 0.14%	0.811	0.099
Gullmars fjord	Sweden	24	7	0.22 ± 0.16%	0.726	0.142

**Table 3:** Pairwise *F*_ST_ values (below the diagonal) and corrected average pairwise differences (above diagonal) among *Symphodus melops* locations calculated from mitochondrial CR sequences. *F*_ST_ bold values indicate significance at *P* < 0.05, after correction using the false discovery rate (FDR). Probability values corrected with Qvalue

	Algarve	Lisbon	Galicia	Roscoff	Plymouth	Belfast	Gullmars fjord	Oslo	Kristiansand	Egersund
Algarve	-	−0.010	−0.029	0.043	0.079	0.006	**2.405**	**2.311**	**2.405**	**2.362**
Lisbon	−0.005	-	−0.026	−0.024	**0.125**	0.049	**2.583**	**2.507**	**2.584**	**2.565**
Galicia	−0.015	−0.016	-	0.002	**0.166**	0.060	**2.668**	**2.587**	**2.670**	**2.634**
Roscoff	0.019	−0.021	−0.003	-	**0.172**	0.089	**2.746**	**2.677**	**2.750**	**2.738**
Plymouth	**0.033**	**0.060**	**0.072**	0.069	-	−0.012	**1.516**	**1.440**	**1.516**	**1.496**
Belfast	0.003	0.028	0.030	0.040	−0.012	-	**1.802**	**1.724**	**1.808**	**1.776**
Gullmars fjord	**0.710**	**0.746**	**0.748**	**0.855**	**0.547**	**0.632**	-	0.002	0.000	0.000
Oslo	**0.680**	**0.721**	**0.721**	**0.818**	**0.515**	**0.598**	0.014	-	0.002	−0.004
Kristiansand	**0.680**	**0.723**	**0.722**	**0.832**	**0.511**	**0.597**	0.001	0.007	-	0.000
Egersund	**0.659**	**0.707**	**0.703**	**0.810**	**0.490**	**0.573**	0.008	−0.020	0.002	-

For the CR data, haplotype diversity values were significantly different among populations according to the χ^2^-test developed by Salicru et al. (1993) (χ^2^ = 152.7, *P* < 0.001). Pairwise comparisons between localities showed that Algarve, Lisbon, Galicia, Roscoff, Plymouth, and Belfast all have significantly higher haplotype diversities than those found in northern locations (Gullmars fjord, Oslo, Kristiansand, Egersund).

SAMOVA (CR dataset) yielded a maximized *F*_CT_ (0.639) for two groups: the four Scandinavian locations versus the six Atlantic locations (*P* = 0.000). Given the concordance between the pairwise *F*_ST_s and SAMOVA, we decided to pool together Egersund, Kristiansand, Oslo, and Gullmar Fjord (Scandinavia) and Plymouth, Belfast, Roscoff, Galicia, Lisbon, and Algarve (Atlantic) for further analyses. An hierarchical AMOVA for the S7 data using the same two groups yielded a relatively low but significant difference among groups and lack of differentiation among populations: percentage of variation among groups = 7. 24 (*P* = 0.010); percentage of variation among populations within groups = – 0.36 (*P* = 0.768); and percentage of variation within populations = 93. 12 (*P* = 0.000) (*F*_ST_ = 0.0688)

Tajima and Fu’*F*s tests, for the CR dataset, yielded negative and significant results both for Scandinavia (Tajima's *D* = –1.936, *P* < 0.001; *F*s = –9.438, *P* < 0.001) and the Atlantic (Tajima's D = –1.977, *P* = 0.006; *F*s = –27.406, *P* < 0.001), both supporting for population expansion. The best fit for the Atlantic was the sudden expansion model (SSD = 0.007, *P* = 0.67), while for Scandinavia was the spatial expansion with constant deme size (SSD = 0.0002, *P* = 0.43). The results of the mismatch analysis showed that the Scandinavian population as a whole displays a very shallow mismatch distribution from zero to two differences, while the Atlantic mismatch distribution is deeper (from 0 to 10 differences).

Using a mutation rate of 5%, we conclude that the estimated age of the Scandinavian population is 2250 years (95% confidence interval: 0–32,800 years), while for the Atlantic the estimated age of the population is about 91,800 years (4010–170,250 years). For the Scandinavian population, the corresponding values for the mutation rates of 3.5% and 10% were 3200 years (0–46,860 years) and 1130 years (0–16,400 years). For the Atlantic population and for the same mutation rates, the numbers varied between 131,160 years (3.5% mutation rate, 5730–243,210 years) and 45,900 years (10% mutation rate, 2000–85,120 years). In summary, although the estimated ages of the expansions vary a lot and have large confidence intervals, the origin of the Scandinavian expansion tends to fall well after the LGM, likely in the current interglacial, while in the Atlantic there are strong indications that the expansion predates the LGM.

**Figure 4:**
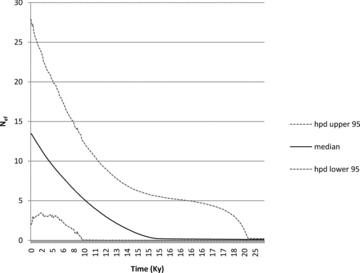
Bayesian skyline plot (BSP) for the control region (CR) dataset of *S. melops* reveals a faster population growth in the past 15,000 years (∼13-fold), reaching an *N*_ef_ of 13 million fish in the present day.

Figure 4 shows the BSP for the CR dataset of *S. melops.* The plot reveals that the corkwing wrasse as a whole experienced a faster population growth in the past 15,000 years (∼13-fold), reaching an *N*_ef_ of 13 million fish in the present day. The *t*_MRCA_ from the Scandinavian population (38,000 years ago) is roughly half of the *t*_MRCA_ obtained for the Atlantic fish (between 60,000 and 74,000 years ago).

Lamarc analyses yielded higher theta for the Atlantic population (average 0.107) in comparison with the Scandinavian (average 0.027). The migration rate was higher northwards, from Atlantic into Scandinavia (average 17.885), than in the opposite direction (average 10.230). We could not perform estimations of population age because the model including growth did not converge.

## Discussion

The present results indicate that although there is considerable homogeneity both within the Atlantic and the Scandinavian samples, there is significant genetic structure among these regions. This separation is evidenced by the topology of the haplotype network (Fig. 2), the *F*_ST_ values (Tables 3 and 4), the SAMOVA outcome (CR), and the hierarchical AMOVA (S7). The intermediate central geographic distribution areas of Plymouth and Belfast contain haplotypes from both south and northern regions, although in very different proportions. The star-shaped topology of the CR haplotype network is consistent with expectations of a recent population expansion (Rogers et al. 1992).

**Table 4:** Pairwise *F*_ST_ values (below the diagonal) and corrected average pairwise differences (above diagonal) among *Symphodus melops* locations calculated from S7 first intron sequences. *F*_ST_ bold values indicate significance at *P* < 0.05, after correction using the false discovery rate (FDR). Probability values corrected with Qvalue

	Algarve	Lisbon	Galicia	Roscoff	Plymouth	Gullmars fjord	Oslo	Kristiansand	Egersund
Algarve	–	−0.017	0.025	0.018	−0.024	**0.362**	**0.324**	**0.373**	**0.345**
Lisbon	−0.011	–	−0.007	−0.018	−0.018	**0.237**	**0.205**	**0.264**	**0.226**
Galicia	0.017	−0.004	–	−0.034	0.008	**0.165**	**0.172**	**2.670**	**0.232**
Roscoff	0.011	−0.013	−0.025	–	0.001	**0.131**	**0.138**	**0.193**	**0.116**
Plymouth	−0.014	−0.010	0.005	−0.002	–	**0.292**	**0.259**	**0.309**	**0.275**
Gullmars fjord	**0.250**	**0.166**	**0.127**	**0.125**	**0.187**	–	0.003	0.023	0.001
Oslo	**0.028**	**0.023**	**0.022**	0.000	**0.030**	−0.001	–	−0.032	0.019
Kristiansand	**0.232**	**0.162**	**0.155**	**0.152**	**0.176**	0.030	−0.014	–	0.054
Egersund	**0.251**	**0.162**	**0.120**	**0.123**	**0.180**	0.001	−0.004	0.080	–

*Symphodus melops* bears a higher diversity in the northeastern Atlantic than in the Scandinavian waters. This pattern would be generated if *S. melops* colonized or recolonized Scandinavia after the last glaciation. The apparent lack of differentiation among Scandinavian populations, the low level of genetic diversity, the scarcity of haplotypes derived from the one that contains most fish, could all be easily explained if the recolonization event was so recent that there was no time for the accumulation of in situ diversification. Within the Atlantic area, the level of genetic diversity remains high across the entire area and displays evidence of persistence of many lineages older than the last glaciation. The mismatch analysis of the pooled samples of the Atlantic area presents lineages differing by 10 mutational steps while the mismatch distribution for the Scandinavia extends only from 0 to 2 mutations. Keeping in mind the notes of caution made in material and methods, on estimating divergence times, it is worth noting that the mismatch analysis provided age estimates that are much younger than the last glaciation for the North Sea (2000 years ago), while for the Atlantic the age estimated is much older than the LGM.

The mismatch analysis supports spatial expansion with constant deme size for the Scandinavian populations and a sudden expansion for the Atlantic populations. The results of BEAST for all samples combined suggest an expansion just after the LGM. Although the LGM took place about 18,000 years ago, several cold episodes such as the Younger Dryas took place until 11–10,000 years ago (e.g., Briggs, 1995). These cold episodes could eliminate the fish fauna of warm temperate origin from most of West Europe. As stated in the introduction, *S. melops* belongs to a genus mostly restricted to the Lusitanian province, with several species confined to the Mediterranean and its surroundings (Froese and Pauly, 2011). Thus, it is likely that *S. melops* is more vulnerable to cold conditions than the more cold adapted boreal fish fauna. For this warm temperate fish, the effects of the last glaciation probably began to fade out only in the last 10,000 years.

We suggest that *S*. *melops* established populations in the North Sea 10,000 years ago or less and had little time to diversify in this northern area. It would be interesting to assess the temperature decreases generated by the Little Ice Age, which occurred around the 17th century (e.g., Mann et al. 2009). Could the environmental conditions during the Little Ice Age be sufficiently severe to eliminate *S. melops* from northern Scandinavia? Paleotemperature reconstructions of this period may allow a proper evaluation of the plausibility of this hypothesis.

Francisco et al. (2009) showed that the sand smelt *Atherina presbyter*, a species with a warm temperate distribution and a few occurrences in the southern North Sea, revealed a phylogeographic pattern similar to the one described here for *S. melops*. A very recent colonization was hypothesized to be the cause of the very reduced genetic diversity observed in the north.

The Mediterranean seems an unlikely candidate as a glacial refugium for *S. melops*. In the Mediterranean, this species is replaced by several other *Symphodus* species, namely its close relative *S. roissali* and its sister species *S. mediterraneus* (Almada et al. 2002; Hanel et al. 2002). The few occurrences of *S. melops* reported in the Mediterranean (Froese and Pauly 2011) apparently do not represent stable populations. During the present study, we contacted fisherman and biologists in the Mediterranean who confirmed the rarity and the sporadic occurrence of the species in that sea.

The Atlantic populations probably survived in situ, at least in the southern part of the species range, from Central Portugal to the south. Indeed, as stated in the introduction, if we compare the paleotemperatures given by CLIMAP (1981) for the western coast of the Iberian Peninsula, we find that at the LGM, temperatures were not lower than they are today in north Scandinavia. Thus, conditions could have remained within what is tolerated by this wrasse species. We suggest that western and southern Iberia could have kept their population of *S. melops*, even if some contraction in population size took place.

As the North Sea was glaciated during the LGM (e.g., CLIMAP 1981), a question arises on the putative refugia of the *S. melops* that recolonized the North Sea when the climate improved. The Atlantic haplotypes are so diverse and spread over an area that is so vast (from Portugal to Belfast and Plymouth) that they could be a potential source of the recolonization of the far north. However, the absence of these haplotypes north of Plymouth and Belfast raises doubts about their role as founders of the new northern populations.

In our view, it seems more likely that the refugium or refugia were located in the vicinity of the North Sea. The Hurd Deep, a marine unglaciated lake in the bottom of the English Channel during the last glaciation, has been postulated as a possible refugium when the surrounding sea was glaciated (e.g., Provan et al. 2005). Other areas around South or Southwest Ireland or Southwest Great Britain were unglaciated and could also have acted as refugia as hypothesized for red and brown algae, *Palmaria palmata* (Provan et al. 2005) and *Fucus serratus* (Hoarau et al. 2007), respectively. They can also have been effective for *S. melops* if the colonization of the North Sea followed a path around western Britain and from Scotland to Skagerrak. Both Belfast and Plymouth show a mixture of Scandinavian and Atlantic haplotypes. It is possible that a finer scale analysis using microsatellites will help to track the pathway of recolonization of northern areas. Is or was it through the English Channel or rather west of United Kingdom reaching Norway through Scotland and surrounding islands? Considering the Scandinavian locations, it would also be very interesting to test if, at present, gene flow takes place between our sampling sites, a task that microsatellites would help to clarify.

To conclude, is it worth comparing our results with those for other inshore organisms of West Europe. *Symphodus melops* stands out, like *A. presbyter* (Francisco et al. 2009), as an example of high diversity in the southern limit and a sharp decline in genetic diversity in the North of the species range. In *A. presbyter*, the eggs are demersal and the young hatch at a rather advanced stage of development, so that a typical larval stage does not exist (Henderson and Bamber, 1987) making the species a good candidate for low dispersal. In contrast, in *L. pholis*, another West European fish whose males guard eggs like those of *S. melops*, the larvae stay a minimum of 29 days in the plankton before settling (Faria et al. 2002). This fish does not display genetic structure along West Europe, the diversity remains at similar levels and the genealogies are rather deep (Francisco et al. 2011).

Although as stated above that the literature has revealed contradictory results, there is an increasing number of papers reporting cases where diversity remains almost at the same level across the latitudinal range of marine species (e.g., *N. norvegicus*, Stamatis et al. 2004; *H. gammarus*, Triantafyllidis et al. 2005; *Petromyzon marinus*, Almada et al. 2008; *S. sprattus*, Debes et al. 2008; *L. pholis*, Francisco et al. 2011). The pioneer studies of terrestrial phylogeography (e.g., Avise 2000; Hewit 2004) postulated that the decline of genetic diversity to the highest latitudes occupied by a species would be the rule, because dispersal through a series of stepping stones would generate a series of bottlenecks that would deplete the variation transported initially from the source population. If, however, in marine organisms with propagules transported in the plankton the numbers of founders are very high and travel long distances, a source population may export much of its diversity to the newly colonized areas. There, if the populations are far from the carrying capacity of the habitat, the bottlenecks may not occur. If, in addition, the dispersal distance per generation continues to be high, as time passes the populations may get mixed, erasing any possible signature of the first colonization. We propose that many marine organisms may display life-history traits conforming to this type of dispersal and colonization. That would explain the lack of a genetic diversity gradient with latitude. If this interpretation is correct, *S. melops* and the already mentioned *A. presbyter* may represent exceptions. They may be very recent arrivals in northern areas or they may display slow dispersal so that a transient gradient in diversity was not yet erased. Further studies with organisms with varied life histories and ecology and population simulations are clearly needed to clarify this issue.
